# Sucralose, a Non-nutritive Artificial Sweetener Exacerbates High Fat Diet-Induced Hepatic Steatosis Through Taste Receptor Type 1 Member 3

**DOI:** 10.3389/fnut.2022.823723

**Published:** 2022-05-23

**Authors:** Hung-Tsung Wu, Ching-Han Lin, Hsiu-Ling Pai, Yi-Cheng Chen, Kai-Pi Cheng, Hsin-Yu Kuo, Chung-Hao Li, Horng-Yih Ou

**Affiliations:** ^1^Department of Internal Medicine, School of Medicine, College of Medicine, National Cheng Kung University, Tainan City, Taiwan; ^2^Division of Endocrinology and Metabolism, Department of Internal Medicine, National Cheng Kung University Hospital, Tainan City, Taiwan; ^3^Graduate Institute of Metabolism and Obesity Sciences, College of Nutrition, Taipei Medical University, Taipei City, Taiwan; ^4^Department of Medical Research, Ditmanson Medical Foundation Chia-Yi Christian Hospital, Chiayi City, Taiwan; ^5^Division of Gastroenterology and Hepatology, Department of Internal Medicine, National Cheng Kung University Hospital, Tainan City, Taiwan; ^6^Institute of Clinical Medicine, College of Medicine, National Cheng Kung University, Tainan City, Taiwan; ^7^Department of Family Medicine, Tainan Municipal An-Nan Hospital, China Medical University, Tainan City, Taiwan

**Keywords:** artificial sweetener, endoplasmic reticulum stress, hepatic steatosis, high fat diet, sucralose

## Abstract

Non-alcoholic fatty liver disease (NAFLD) is the most common chronic liver disease globally, and it is strongly associated with obesity. To combat obesity, artificial sweeteners are often used to replace natural sugars, and sucralose is one of the most extensively used sweeteners. It was known that sucralose exerted effects on lipid metabolism dysregulation, and hepatic inflammation; however, the effects of sucralose on hepatic steatosis were still obscure. In this study, we found that supplements of sucralose enhanced high-fat-diet (HFD)-induced hepatic steatosis. In addition, treatment of sucralose increased reactive oxygen species (ROS) generation and induced endoplasmic reticulum (ER) stress in HepG2 cells. Pretreatment of ROS or ER stress inhibitors reversed the effects of sucralose on lipogenesis. Furthermore, pretreatment of taste receptor type 1 membrane 3 (T1R3) inhibitor or T1R3 knockdown reversed sucralose-induced lipogenesis in HepG2 cells. Taken together, sucralose might activate T1R3 to generate ROS and promote ER stress and lipogenesis, and further accelerate to the development of hepatic steatosis.

## Introduction

Non-alcoholic fatty liver disease (NAFLD) is the most common chronic liver disease globally, and it is strongly associated with metabolic diseases, such as obesity and type 2 diabetes (1, 2). NAFLD is defined as ectopic lipid accumulation in the liver and encompasses a spectrum of disease severity ranging from simple steatosis, non-alcoholic steatohepatitis, and cirrhosis to hepatocellular carcinoma. Increased hepatic lipid accumulation results in reactive oxygen species (ROS) generation and is associated with mitochondrial dysfunction, and endoplasmic reticulum (ER) stress (3), leading to the development of an impaired liver function.

To combat obesity and further decrease the prevalence of metabolic diseases, including NAFLD, sweeteners with the characteristic of high-intensity sweetness and low calories are used to control the body weight in obese subjects. Among the artificial sweeteners, sucralose is extensively used due to pH and heat stability, and has been declared safe by the Food and Drug Administration (FDA) in the United States. However, several studies indicated that sucralose increased the expressions of hepatic pro-inflammatory factors (4), induced hepatic lymphocytic infiltration (5), and increased rat hepatic lipogenesis-related gene levels (6). Sucralose also altered bile acid metabolism (7) and glucose tolerance (8) by changing the composition of gut microbiota. Recent evidence suggested that sucralose had an activity to induce ER stress pathway (9). Although sucralose was thought to be metabolically inactive (10), these studies indicated that sucralose may exert adverse effects on human health.

The sweet taste receptors (STR), including taste receptor type 1 membrane 2 (T1R2) and taste receptor type 1 membrane 3 (T1R3) are expressed in numerous extra-gustatory tissues (11), playing an important role in energy balance, glucose homeostasis (12), and lipid metabolism (13). It was known that STR are also expressed in liver (14, 15), and loss of T1R3 reduces atherosclerotic plaque accumulation and hepatic steatosis in ApoE knockout mice (16). Although STR can be activated by sucralose and further triggering of physiological responses, the effects of sucralose on hepatic lipid accumulation were still obscure.

To investigate the effects of sucralose on hepatic steatosis in obesity, we used high-fat-diet (HFD)-fed mice and HepG2 cells to evaluate the effects of sucralose on hepatic lipid accumulation and clarified the possible mechanisms in this study.

## Materials and Methods

### Animals

All animal experiments were approved by the Institutional Animal Care and Use Committee of Taipei Medical University (LAC-2020-0302) and performed as National Institutes of Health guide for the care and use of laboratory animals. Male C57BL/6 mice were purchased from National Laboratory Animal Center (Taipei, Taiwan) and housed in a temperature (23 ± 2°C) and humidity (60 ± 10%) control environment. The mice at the age of 8-week were randomly divided into three groups, and received the treatments for 12 weeks: (1) mice fed with a chow diet (5001, LabDiet, St. Louis, MO, United States) (Chow group); (2) mice fed with high-fat diet (58Y1, 60% kcal from fat, TestDiet, St. Louis, MO, United States) (HFD group); and (3) HFD supplemented 500 ppm sucralose (Alfa Aesar™, Tewksbury, MA, United States) (HFSUC group). The dose of sucralose used in this study was followed by a previous report that showed that there were no adverse effects at a dose of 500 ppm sucralose supplement (17). The body weight, daily food, and water intake were recorded every week using metabolic cages (Ugo Basile, Gemonio, Italy). At the end of the experiments, all groups of the mice were fasted for 8 h and then well-anesthetized to sacrifice. The liver tissue and epididymal fat pads (eWAT) were removed and weighed. In addition, the left hepatic lobe tissue of each group of the mice was cut into pieces and then fixed in 10% formalin or frozen at −80°C for further experiments. Fixed specimens were dehydrated and embedded in paraffin. The block was then cut into 5-μm-thick sections and stained with hematoxylin and eosin (H&E). The sections were observed with a light microscope (Olympus, Tokyo, Japan).

### Determination of Hepatic Triglyceride Content

Thirty mg of left hepatic lobe tissues of the mice was homogenized with phosphate-buffered saline (PBS), and a triglyceride colorimetric assay kit (Cayman Chemical) was used to determine the hepatic triglyceride content. Alanine aminotransferase (ALT) and aspartate aminotransferase (AST) activities in the liver were measured by ALT and AST liquid reagents (Teco Diagnostics, Anaheim, CA, United States), respectively.

### Cell Culture

Hepatocellular carcinoma HepG2 and human embryonic kidney 293T (HEK293T) cell lines were purchased from Bioresource Collection and Research Center (Food Industry Research and Development Institute, Hsinchu, Taiwan). The cells were cultured in high-glucose Dulbecco’s Modified Eagle Medium (DMEM; Hyclone, South Logan, UT, United States) with 10% fetal bovine serum (Hyclone) and 1% of penicillin and streptomycin at 37°C in 5% CO_2_.

### Lentiviral Vectors

Lentiviral vectors containing short hairpin ribonucleic acid (shRNA) targeted to T1R3 were purchased from the RNAi Core Facility (Academia Sinica, Taipei, Taiwan) to knockdown T1R3 as previously described (18). Briefly, lentiviral vectors expressing shRNA were produced by the transfection of HEK293T cells with the lentiviral transfer plasmid along with psPAX2 and pMD2G plasmids. At 48 h after transfection, the supernatant from the cultures was collected, filtered through 45-μM pore-size filters, and used to infect HepG2 cells, and 2 μg/ml puromycin was used for the selection. The clone of HepG2 cells with the greater knockdown of T1R3 levels, determined by Western blots, was used for further experiments.

### Analysis of Lipid Content and Cellular Triglyceride Measurement

Lipid accumulation in HepG2 cells was observed by Oil Red O staining (Sigma-Aldrich, St. Louis, MO, United States). The cells were cultured at a density of 2 × 10^5^ cells/10 cm dish and pretreated with 0.1, 1, and 10mM sucralose with low glucose DMEM. After 24 h, 0.5 mM oleic acid (OA; Sigma-Aldrich) chelated with 2% bovine serum albumin (Sigma-Aldrich) was then added to each group of the treatment for another 24 h. The cells were washed three times with PBS and fixed with 4% paraformaldehyde for 5 min. After fixation, cells were washed with 60% isopropanol and stained with Oil Red O at room temperature for 15 min. Cellular lipid accumulation was observed using a light microscope (Olympus).

To determine cellular lipid content, the cells were shocked for 30 min and simultaneously centrifuged at 12,000 rpm for 15 min, and the supernatant was collected. Ten μl of the supernatant was used for protein quantification by the bicinchoninic acid (BCA) protein assay kit (Visual Protein, Taipei, Taiwan), and the remaining supernatant was extracted by methanol:chloroform = 2:1 according to a previous study (19). Cellular triglycerides were quantified with a commercialized assay kit (Cayman Chemical), according to the manufacturer’s instructions and normalized to total protein concentration.

### Detection of Cellular Reactive Oxygen Species Generation

Cellular ROS generation was measured using 2′, 7′-dichlorodihydrofluorescein diacetate (DCFDA, Invitrogen, Austin, TX, United States). HepG2 cells were cultured in a 6 cm dish at a density of 2 × 10^5^ cells/dish and pretreated with gymnemic acid (GA, Taiclone, Taipei, Taiwan) or lactisole (Cayman Chemical) for 30 min. After the treatment of 10 mM sucralose for another 90 min, cells were stained by 10 μM DCFDA for 30 min at 37°C in dark. Then, cells were washed with warm PBS three times and then observed using a fluorescence microscope (Olympus). The cellular ROS generation was quantified by fluorescence intensity using image analytical software (Image J; National Institutes of Health, Bethesda, MD, United States).

### Quantitative Real-Time-Polymerase Chain Reaction

The total RNA of HepG2 cells treated with sucralose was isolated using REzol™ reagent (Protech Technology, Taipei, Taiwan). A total of 2 μg RNA was reverse-transcripted by the Moloney murine leukemia virus reverse transcription kit containing 1 mM deoxynucleotide triphosphate, 40 U/μl RNase inhibitor, and 1 mM random hexamer primers (Protech Technology), in a total volume of 20 μl, and incubated for 1 h at 42°C and 10 min at 70°C. Quantitative real-time-polymerase chain reaction (qPCR) was performed in a final of 20 μl containing 100 ng sample cDNA, 5 μM forward and reverse primers, and 10 μl Luna^®^ Universal qPCR Mix (New England Biolabs, Ipswich, MA, United States). The qPCR was performed using StepOnePlus Real-Time PCR System (Applied Biosystems, Waltham, MA, United States), according to the manufacturer’s instructions. The RNA expressions were normalized to GAPDH by using the 2^–ΔΔCt^ method.

### Western Blot Analysis

The protein samples of HepG2 cells or left hepatic lobe tissues of the mice were extracted using a radioimmunoprecipitation lysis buffer (VWR Chemicals, Solon, OH, United States) with protease and phosphatase inhibitors (MedChemExpress, Monmouth Junction, NJ, United States). After centrifugation at 13,000 rpm for 10 min at 4°C, the supernatant was collected for the determination of protein concentrations by the BCA assay. Samples containing 30 μg of protein were separated by sodium dodecyl sulfate-polyacrylamide gel electrophoresis and then transferred to polyvinylidene difluoride membranes (Biomate, Taipei, Taiwan). The membranes were blocked with 10% skim milk in TBS-T [20 mM Tris (pH 7.4), 150 mM NaCl, and 0.1% Tween 20] for 1 h at room temperature and then incubated with primary antibodies, such as carbohydrate-responsive element-binding protein (ChREBP; Novus Biologicals, Littleton, CO, United States), sterol regulatory element-binding transcription factor-1 (SREBP1; Novus Biologicals), fatty acid synthase (FASN; Abcam, Cambridge, United Kingdom), acetyl-CoA carboxylase (ACC; Cell Signaling, Danvers, MA, United States), inositol requiring enzyme-1α (IRE1α; Cell Signaling), X-box binding protein-1 (XBP1; Affinity, Golden, CO, United States), T1R2 (Invitrogen), T1R3 (LifeSpan BioSciences, Seattle, WA, United States), peroxisome proliferator-activated receptor γ (PPARγ; Abcam), and actin (Millipore, Burlington, MA, United States) at 4°C overnight. The blots were then washed with TBS-T buffer and incubated with horseradish peroxidase-conjugated secondary antibodies at room temperature for 1 h. The blots were detected by using enhanced chemiluminescence (Millipore), and the relative signal intensity was quantified using ImageJ software.

### Statistics

GraphPad Prism 6 was used for all statistical analyses. Data were expressed as mean ± standard error (SEM). Statistical analysis was conducted using Student’s *t*-test or one-way ANOVA followed by Tukey’s *post hoc* test. A value of *p* < 0.05 was considered statistically significant.

## Results

### Sucralose Exacerbated High-Fat-Diet-Induced Hepatic Steatosis in Mice

**FIGURE 1:**
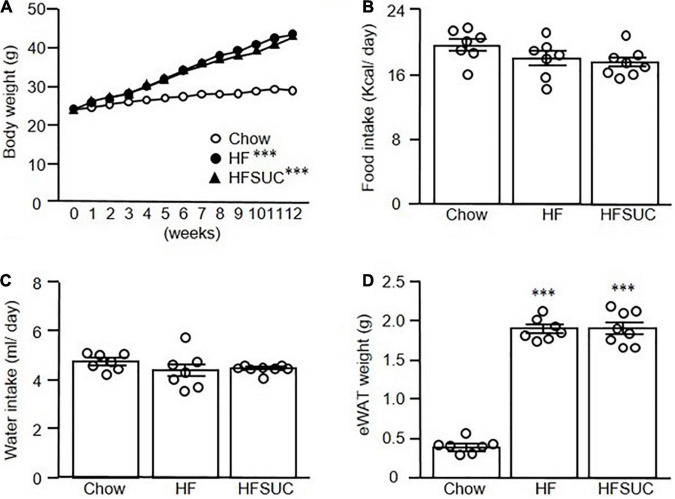
Supplemen of sucralose in high-fat diet showed no significant effects on the body weight of mice. Eight-week-old C57BL/6 mice were fed with a chow diet (Chow), high-fat diet (HFD), or HFD supplemented with sucralose (HFSUC) for 12 weeks, and the body weight **(A)**, food intake **(B)**, and water intake **(C)** were measured using metabolic cages. At the end of the experiments, each group of the mice was sacrificed and the epididymal fat pads (eWAT) were removed and weighed **(D)**. Data are mean ± SEM (*n* = 7–8 for each group of the mice) ^***^p < 0.001 as compared with the Chow group.

**FIGURE 2:**
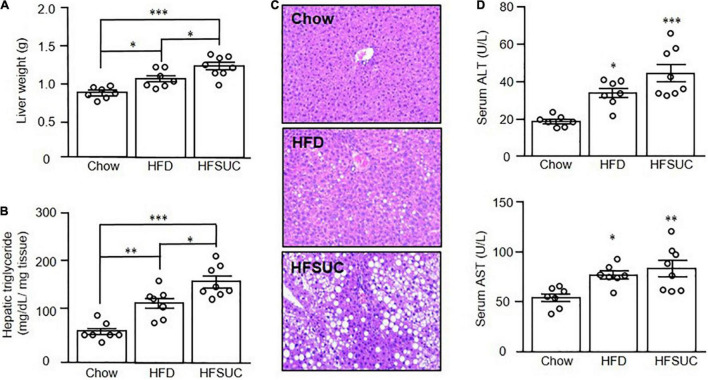
Supplement of sucralose exacerbated high fat diet-induced hepatic steatosis in mice. Eight-week-old C57BL/6 mice were fed with a chow diet (Chow), high-fat diet (HFD), or HFD supplemented with sucralose (HFSUC) for 12 weeks. At the end of the experiments, each group of the mice was sacrificed and the liver tissues were removed and weighed **(A)**. Hepatic triglyceride contents for each group of the mice were measured using a commercialized assay kit **(B)**. In addition, the liver sections were stained with hematoxylin and eosin (100X) **(C)**. Serum samples were collected for the determination of alanine aminotransferase (ALT), and aspartate aminotransferase (AST) levels **(D)**. Data are mean ± SEM (*n* = 7–8 for each group of the mice) *p < 0.05, ^**^p < 0.01, ^***^p < 0.001 as compared with the Chow group or indicated groups.

After a 12-week feeding of HFD (HFD group) or HFD supplement with sucralose (HFSUC group), the bodyweight of both groups was significantly increased as compared with the Chow group, whereas there were no differences between the HFD and HFSUC groups (Figure 1A). In addition, the daily calorie intake (Figure 1B) and water intake (Figure 1C) showed no significant differences among the three groups. Also, the eWAT weight of the HFD and HFSUC groups were significantly increased, as compared with the Chow group (Figure 1D). However, the liver weight of the HFSUC group was significantly higher than the HFD group (Figure 2A). We found that the hepatic triglyceride levels (Figure 2B), and lipid droplets accumulation (Figure 2C) were significantly increased in the HFSUC group, as compared with the HFD group. Furthermore, plasma ALT and AST levels were significantly increased in the HFD group. When given the supplement of sucralose, a trend of increased ALT and AST levels were observed, as compared with the HFD group (Figure 2D). These data suggested that sucralose might exacerbate high fat diet-induced NAFLD.

### Sucralose Induced Hepatic Endoplasmic Reticulum Stress to Increase Lipogenesis-Related Protein Expressions in Mice

**FIGURE 3:**
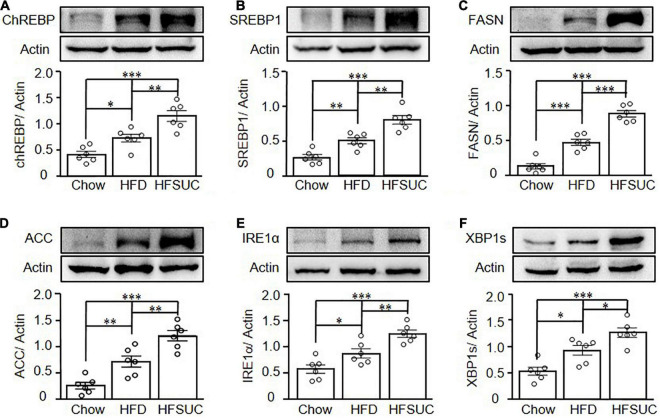
Sucralose increased hepatic ER stress- and lipogenesis-related protein expressions in mice. Eight-week-old C57BL/6 mice were fed with a chow diet (Chow), high-fat diet (HFD), or HFD supplemented with sucralose (HFSUC) for 12 weeks. At the end of the experiments, each group of the mice was sacrificed and the liver tissues were removed. Western blots analysis was used to analyze the hepatic expressions of lipogenesis-related proteins, carbohydrate-response element-binding protein (ChREBP) **(A)**, sterol regulatory element-binding protein 1 (SREBP1) **(B)**, fatty acid synthase (FASN) **(C)**, and acetyl-CoA carboxylase 1 (ACC1) **(D)**, and ER stress-related proteins, IRE1α **(E)**, and XBP1s **(F)**. Data are mean ± SEM (*n* = 6 for each group) *p < 0.05, ^**^p < 0.01, ^***^p < 0.001 as compared with indicated groups.

Since the effects of sucralose on hepatic steatosis were observed in mice, we then evaluated the levels of lipogenesis protein expressions in the liver, such as ChREBP (Figure 3A), SREBP1 (Figure 3B), FASN (Figure 3C), and ACC (Figure 3D). We found that the hepatic lipogenesis-related proteins were significantly increased in the HFD group as compared with the Chow group. Supplement of sucralose in HFD further enhanced the levels of hepatic lipogenesis proteins (Figures 3A–D).

It was known that ER stress is one of the key pathways that induces the development of NAFLD. Activation of IRE1 and XBP1 was known to be induced by ER stress and participated in hepatic lipogenesis. Thus, we then further investigated the effects of sucralose on ER stress in the livers of mice. We found that the expressions of IRE1α and spliced XBP1 (XBP1s) were significantly increased in the HFD group as compared with the Chow group. In addition, sucralose supplement further significantly enhanced the expressions of IRE1α (Figure 3E) and XBP1s (Figure 3F) in the HFSUC group.

### Sucralose Promoted Lipogenesis to Increase Lipid Accumulation in HepG2 Cells

**FIGURE 4:**
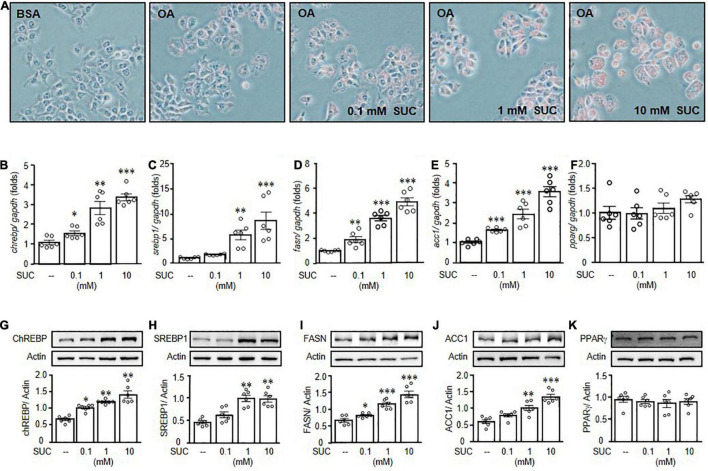
Sucralose increased lipogenesis to facilitate lipid accumulation in HepG2 cells. HepG2 cells were pretreated with indicated doses of sucralose (SUC) for 24 h, and then treated with 0.5 mM oleic acid (OA) chelated with 2% bovine serum albumin (BSA) for another 24 h. Oil Red O staining was used to detect lipid accumulation (original magnification 200X) **(A)**. In addition, the gene **(B–F)**, and protein **(G–K)** expressions of carbohydrate-response element-binding protein (ChREBP), sterol regulatory element-binding protein 1 (SREBP1), fatty acid synthase (FASN), and acetyl-CoA carboxylase 1 (ACC1) were analyzed by real-time polymerase chain reaction and Western blots. Data are mean ± SEM (*n* = 6 for each group) *p < 0.05, ^**^p < 0.01, ^***^p < 0.001 as compared with control group.

To investigate the possible mechanisms of sucralose-induced hepatic steatosis, HepG2 cells were used, and treated with different doses of sucralose to evaluate whether sucralose had an effect to accelerate oleic acid (OA)-induced lipid accumulation. Consistent with the observations in animals, sucralose dose-dependently enhanced OA-induced lipid accumulation, determined by Oil Red O staining (Figure 4A). In addition, sucralose significantly elevated lipogenesis-related gene expressions, such as *ChREBP* (Figure 4B), *SREBP1* (Figure 4C), *FASN* (Figure 4D), and *ACC1* (Figure 4E). However, the expression of *PPARG* showed no significant changes after sucralose treatment (Figure 4F). Consistent with the observations in mRNA expressions, the protein levels of ChREBP (Figure 4G), SREBP1 (Figure 4H), FASN (Figure 4I), and ACC (Figure 4J) were significantly increased in HepG2 cells after sucralose treatment in a dose-dependent manner, whereas sucralose showed no significant effects on the PPARγ expression (Figure 4K). These results implied that sucralose might induce lipogenesis-related protein expressions to increase hepatic steatosis.

### Sucralose Induced Lipogenesis Through ROS-IRE1-XBP1 Pathway in HepG2 Cells

**FIGURE 5:**
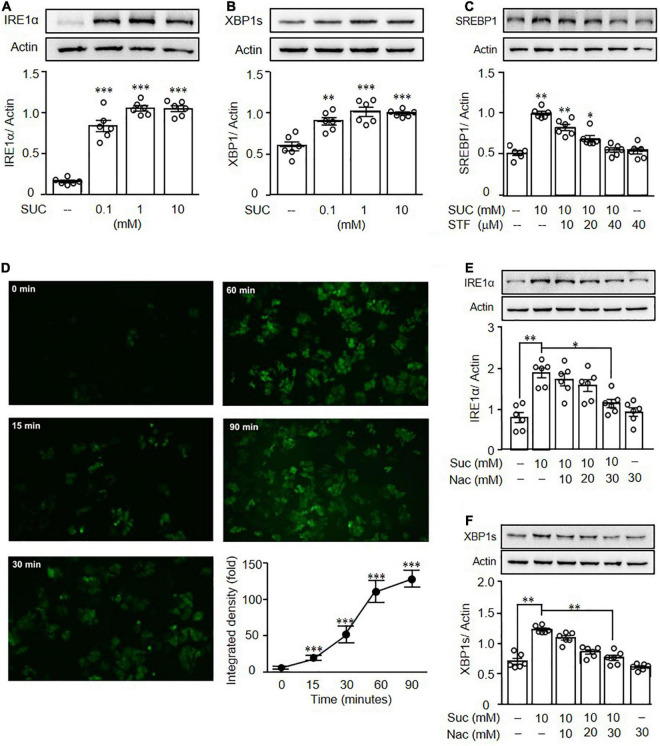
Sucralose increased reactive oxygen species to induce endoplasmic reticulum stress in HepG2 cells. HepG2 cells were treated with indicated concentrations of sucralose (SUC) for 16 h to analyze the expressions of IRE1α **(A)** and XBP1s **(B)** by Western blots. The cells were pretreated with STF083010 (IRE1 inhibitor) at indicated doses for 30 min, and then treated with 10 mM sucralose for another 24 h for the determination of SREBP1 by Western blots **(C)**. HepG2 cells were treated with 10 mM sucralose and the reactive oxygen species production was determined by DCFDA staining at indicated times. The fluorescence intensity was analyzed by ImageJ software (100X) **(D)**. Cells were pretreated with N-acetylcysteine (NAC) at indicated doses for 30 min, and then treated with 10 mM sucralose for another 16 h to determine IRE1α **(E)** and XBP1s **(F)** expressions by Western blots. Data are the mean ± SEM (*n* = 6 for each group) *p < 0.05, ^**^p < 0.01, ^***^p < 0.001 as compared with indicated groups.

To determine the underlying mechanisms of sucralose-induced hepatic steatosis, we further investigated the effect of ER stress on sucralose-induced lipogenesis in HepG2 cells. In addition, it was known that activation of the IRE1-XBP1 pathway is associated with liver diseases (20). Treatment of sucralose significantly increased the expressions of IRE1α (Figure 5A) and XBP1s (Figure 5B) in HepG2 cells. It was known that hepatic IRE1 expression was significantly increased in mice fed with HFD (21). IRE1 inhibitor, STF083010 has an effect to block IRE1 endonuclease activity without affecting its kinase activity under ER stress. We therefore used STF083010 to evaluate the role of IRE1 in sucralose-induced hepatic lipogenesis (22). As shown in Figure 5C, pretreatment of STF083010 reversed the effects of sucralose on the expression of SREBP1, indicating sucralose-induced hepatic lipogenesis through the IRE1-XBP1 pathway.

It was known that sucralose promotes ROS generation to increase adipogenesis in mesenchymal stromal cells (23). Thus, we then investigated whether sucralose induced ER stress through ROS production. As shown in Figure 5D, sucralose significantly increased intracellular ROS levels in a time-dependent manner in HepG2 cells. Pretreatment ROS scavenger, N-acetyl-cysteine (NAC) reversed the effects of sucralose on IRE1α (Figure 5E) and XBP1s (Figure 5F), indicating sucralose generated ROS production to induce ER stress in HepG2 cells.

### Sucralose Increased Hepatic Lipogenesis Through T1R3

**FIGURE 6:**
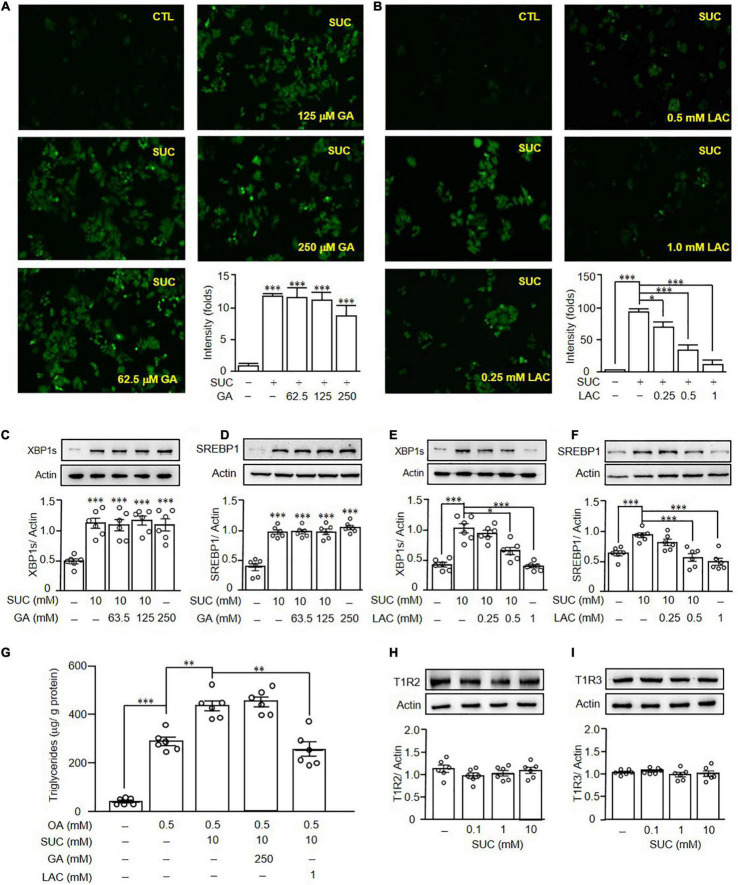
Sucralose induced reactive oxygen species generation and lipogenesis through T1R3. HepG2 cells were pretreated with gymnemic acid (GA) **(A)** or lactisole (LAC) **(B)** at indicated doses for 30 min, and then treated with 10 mM sucralose (SUC) for another 90 min. The reactive oxygen species production was determined by DCFDA staining, and the fluorescence intensity was analyzed by ImageJ software (original magnification 100X). HepG2 cells were pretreated with GA **(C,D)** or LAC **(E,F)** at indicated doses for 30 min, and then treated with 10 mM sucralose for another 24 h to determine XBP1s, and SREBP1 expressions by Western blots. In addition, the cells were harvested to determine the intracellular triglyceride contents using a commercialized assay kit **(G)**. The cells were treated with 10 mM sucralose for 24 h to determine T1R2 **(H)** and T1R3 **(I)** expressions by Western blots. Data are mean ± SEM (*n* = 6 for each group) *p < 0.05, ^**^p < 0.01, ^***^p < 0.001 as compared with indicated groups.

**FIGURE 7:**
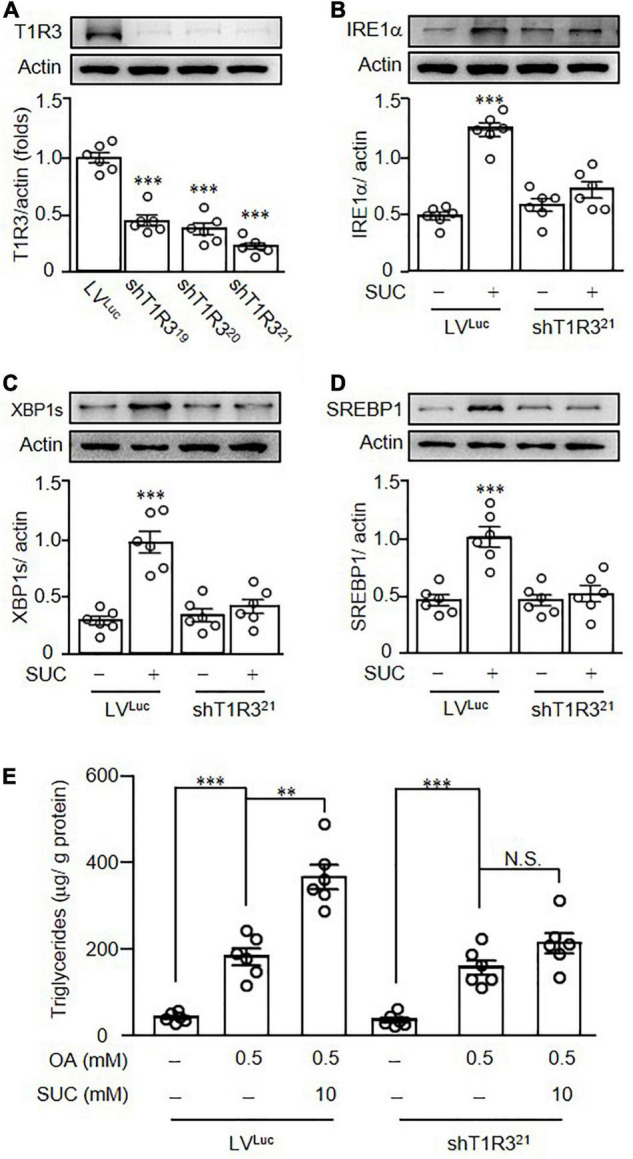
Knockdown of T1R3 in HepG2 cells diminished the effects of sucralose on lipogenesis. Different clones of lentiviral vectors containing short hairpin RNA targeted to T1R3 (shT1R3) were used to evaluate the knockdown efficiency of T1R3 **(A)**. HepG2 cells transfected with lentiviral vectors containing luciferase (LV*^LUC^*) or short hairpin RNA targeted to T1R3 (shT1R3^21^) were treated with 10 mM sucralose (SUC) for 24 h to determine IRE1α **(B)**, XBP1s **(C)**, and SREBP1 expressions **(D)** by Western blots. In addition, HepG2 cells transfected with lentiviral particles were pretreated with 10 mM sucralose for 24 h, and then treated with 0.5 mM oleic acid (OA) for another 24 h. Cells were harvested and the intracellular triglyceride contents using a commercialized assay kit **(E)**. ^**^p < 0.01, ^***^p < 0.001 as compared with LV*^LUC^* group or indicated groups. N.S., no significance.

It was known that sucralose is a ligand for STR, and activation of STR induced ROS production (24), we therefore investigated the role of STR in sucralose-induced lipogenesis. Pretreatment of gymnemic acid (GA, T1R2 inhibitor) showed no significant effects on sucralose-induced ROS production (Figure 6A). On the other hand, pretreatment of lactisole (LAC, T1R3 inhibitor) dose-dependently reversed the effects of sucralose on ROS production (Figure 6B), indicating sucralose increased ROS levels through T1R3. Consistent with the observations in ROS production, pretreatment of GA had no significant effects on both sucralose-induced XBP1s (Figure 6C), and SREBP1 (Figure 6D) expressions, whereas pretreatment of LAC declined the effect of sucralose on the increment in XBP1s (Figure 6E), and SREBP1 (Figure 6F). Also, sucralose-enhanced lipid accumulation was blocked by the T1R3 inhibitor in OA-treated HepG2 cells (Figure 6G). Furthermore, we found that sucralose treatment showed no significant effects on the expressions of T1R2 (Figure 6H), and T1R3 (Figure 6I). To confirm the role of T1R3 in sucralose-induced lipogenesis, lentiviral vectors containing shRNA targeted to T1R3 were used. As shown in Figure 7A, knockdown of T1R3 was successfully achieved by using the lentiviral particles, and clone 21 was the most effective among these vectors. We found the effect of sucralose on the expressions of IRE1α (Figure 7B), XBP1s (Figure 7C), and SREBP1 (Figure 7D), as well as sucralose-enhanced OA lipid accumulation (Figure 7E) were diminished after knockdown of T1R3 in HepG2 cells. These results suggested that sucralose might bind to T1R3 to activate the downstream signals and further promote lipogenesis in the liver.

## Discussion

To the best of our knowledge, this is the first study reported that sucralose consumption might exacerbate HFD-induced NAFLD. It was known that the accumulation of lipid droplets increased ROS production and induced ER stress in hepatocytes (25). In addition, sucralose promotes ROS generation in mesenchymal stromal cells (23), and sucralose-induced hypothalamic ER stress gene expressions were reported (9). However, the effects of sucralose-induced ROS production and ER stress in the liver have not been studied. In this study, we found that sucralose increased lipogenesis through T1R3-ROS-ER stress-dependent pathway.

In this study, the daily caloric intake of C57BL/6 mice was ∼20 Kcal/day/per mouse consistent with a previous study (26). In addition, the mice were fed *ad libitum* in this study, and the satiety center in the brain had an effect to avoid the animals from the intake of excessive calories. Thus, the daily caloric intake of the mice showed no significant differences among the three groups. In addition, C57BL/6 is an obesity-prone strain of the mice, and is more sensitive to HFD (27). Thus, the high fat- and sucralose-treated animals were fatter even if they were consuming the same calories as the controls. In addition, different species of the mice showed variable degrees of susceptibility to HFD (28). Although a previous study indicated that *de novo* lipogenesis was suppressed in BDF1 mice fed with HFD (29), a number of the studies indicated that HFD-induced hepatic lipogenesis in C57BL/6 mice (30–32). In addition to the different species of the mice used in various studies, the composition of the HFD differs from the studies might also be a factor that affects the expression of lipogenesis-related genes.

Several studies revealed the effects of sucralose on the gastrointestinal tract, pancreas, and adipose tissues (33, 34), whereas few pieces of research focused on the liver. Although it was thought that the supplement of sucralose in foods is safe for human health; however, a previous study indicated that maternal exposure to sucralose altered offspring’s gut microbiota and increased hepatic steatosis in adulthood (35). In addition, it was found that long-term sucralose intake increased blood low-density lipoprotein cholesterol (36), hepatic total cholesterol (7), fatty acids (37), and lipogenesis-related genes levels (6). Consistent with previous studies, we found that sucralose enhanced lipid accumulation and markedly elevated the levels of lipogenesis-related proteins in both the cell and animal models.

Although palmitate is the most abundant fatty acid in the human body and a predominant fatty acid in the diet, previous studies indicated that unsaturated fatty acids, such as OA were more steatogenic than saturated fatty acids, such as palmitate (38, 39). It was believed that unsaturated fatty acids were easily esterified into triglycerides and further protected cells from free fatty acids-induced ROS generation (40). Thus, we used OA to evaluate the effects of sucralose on hepatic steatosis.

The expressions of STR were found in extra-gustatory tissues, and STR has various physiological functions in different tissues. However, the role of STR in the liver was still obscure. A recent study revealed that STR was involved in ROS production (24). Pretreatment of lactisole, a T1R3 inhibitor, decreased sucralose-induced ROS levels and also reduced hepatic lipogenesis. These data suggested that sucralose promoted ROS production through T1R3, and then facilitated hepatic lipogenesis.

Taken together, our results demonstrated that sucralose might exacerbate HFD-induced fatty liver. FDA-approved acceptable daily intake (ADI) for sucralose in humans is 5 mg/kg (41). The dose used in our study was 500 ppm in the diet, after multiplying by the ratio of mouse *km* factor and human *km* factor (42), the human equivalent dose approximately equals ADI. Thus, recommendations are needed for the use of artificial sweeteners for weight reduction in the management of NAFLD.

## Data Availability Statement

The original contributions presented in the study are included in the article/supplementary material, further inquiries can be directed to the corresponding author.

## Ethics Statement

The animal study was reviewed and approved by the Institutional Animal Care and Use Committee of Taipei Medical University.

## Author Contributions

C-HLin, H-LP, and Y-CC designed and performed the experiments, analyzed the data, and wrote the manuscript. K-PC and H-YK wrote the manuscript and edited the text. C-HLi provided technical assistance. H-TW and H-YO designed and supervised the study and wrote the manuscript. All authors have read and agreed to the published version of the manuscript.

## Conflict of Interest

The authors declare that the research was conducted in the absence of any commercial or financial relationships that could be construed as a potential conflict of interest.

## Publisher’s Note

All claims expressed in this article are solely those of the authors and do not necessarily represent those of their affiliated organizations, or those of the publisher, the editors and the reviewers. Any product that may be evaluated in this article, or claim that may be made by its manufacturer, is not guaranteed or endorsed by the publisher.
